# Efficacy of andrographolide in not active progressive multiple sclerosis: a prospective exploratory double-blind, parallel-group, randomized, placebo-controlled trial

**DOI:** 10.1186/s12883-020-01745-w

**Published:** 2020-05-07

**Authors:** Ethel Ciampi, Reinaldo Uribe-San-Martin, Claudia Cárcamo, Juan Pablo Cruz, Ana Reyes, Diego Reyes, Carmen Pinto, Macarena Vásquez, Rafael A. Burgos, Juan Hancke

**Affiliations:** 1 Neurology Department, Faculty of Medicine, Pontificia Universidad Católica de Chile, Diagonal Paraguay, 362, 5° floor, Santiago, Chile; 2 Neurology, Hospital Dr. Sótero del Río, Av. Concha y Toro, 3459 Santiago, Chile; 3 Radiology, Pontificia Universidad Católica de Chile, Diagonal Paraguay, 362, 5° floor, Santiago, Chile; 4 Pharmacology and Morphophysiology, Faculty of Veterinary Sciences, Universidad Austral de Chile, Independencia, 613 Valdivia, Chile

**Keywords:** Multiple sclerosis, Progressive multiple sclerosis, Andrographolide, Brain atrophy, Disability progression

## Abstract

**Background:**

Multiple sclerosis (MS) is a chronic immune mediated disease and the progressive phase appears to have significant neurodegenerative mechanisms. The classification of the course of progressive MS (PMS) has been re-organized into categories of active vs. not active inflammatory disease and the presence vs. absence of gradual disease progression. Clinical trial experience to date in PMS with anti-inflammatory medications has shown limited effect. Andrographolide is a new class of anti-inflammatory agent, that has been proposed as a potential drug for autoimmune disorders, including MS.

In the present trial, we perform an exploratory pilot study on the efficacy and safety of andrographolide (AP) compared to placebo in not active PMS.

**Methods:**

A pilot clinical trial using 140 mg oral AP or placebo twice daily for 24 months in patients with not active primary or secondary progressive MS was conducted. The primary efficacy endpoint was the mean percentage brain volume change (mPBVC). Secondary efficacy endpoints included 3-month confirmed disability progression (3-CDP) and mean EDSS change.

**Results:**

Forty-four patients were randomized: 23 were assigned to the AP group, and 21 were assigned to the placebo group. The median baseline EDSS of both groups was 6.0. Annualized mPBVC was − 0.679% for the AP group and − 1.069% for the placebo group (mean difference: -0.39; 95% CI [− 0.836–0.055], *p* = 0.08, relative reduction: 36.5%). In the AP group, 30% had 3-CDP compared to 41% in the placebo group (HR: 0.596; 95% CI [0.200–1.777], *p* = 0.06). The mean EDSS change was − 0.025 in the AP group and + 0.352 in the placebo group (mean difference: 0.63, *p* = 0.042). Adverse events related to AP were mild rash and dysgeusia.

**Conclusions:**

AP was well tolerated and showed a potential effect in reducing brain atrophy and disability progression, that need to be further evaluated in a larger clinical trial.

**Trial registration:**

ClinicalTrials.gov NCT02273635 retrospectively registered on October 24th, 2014.

## Background

Progressive multiple sclerosis (PMS) comprises two clinical phenotypes that depend upon the initial disease course: primary (gradual worsening from onset) and secondary (after a period consistent with relapsing-remitting disease). Additionally, sub-classification according to clinical relapses or radiological activity (active or not active phenotypes) and with or without progression of disability, has been proposed to better define and select patients who would benefit from starting or switching to higher efficacy disease-modifying therapies [1].

Although some pathological differences in the not active progressive phenotypes have been found (e.g. profound axonal loss, moderate inflammation and axonal injury), the pathological findings show similar hallmarks, including slowly expanding lesions, cortical demyelination, diffuse white and grey matter injury, and ectopic meningeal lymphoid tissue [2]. Chronic inflammation is usually compartmentalized within the central nervous system in late disease (e.g. meningeal infiltrates and B cells), and neurodegeneration, although occurring from early stages, is perpetuated by microglial activation, mitochondrial dysfunction and oxidative injury [3, 4]. Therapeutic drugs with anti-inflammatory and immunosuppressive properties, such as ocrelizumab [5] and siponimod [6], have been recently approved for primary and secondary PMS, respectively, which represents an advancement in MS treatment. Between 20 and 25% of the patients in both of the above trials had baseline magnetic resonance imaging (MRI) that showed gadolinium-enhancing lesions (active PMS), however, the subgroup analysis was not powered to show between-group differences among these subgroups.

Since the considerable burden of MS-related symptoms and the need to identify effective interventions to prevent disease progression, various standardized herbal medicinal products, herbal preparations and isolated natural compounds, have been tested in clinical trials as complementary treatments [7].

Andrographolide is a bicyclic diterpenoid lactone isolated from *Andrographis paniculata*, a medicinal herb from Southeast Asia that has been used for the treatment of common colds, fever, laryngitis, rheumatoid arthritis and osteoarthritis and other infections with no or minimal side effects [8–10]. Andrographolide (AP) can be particularly effective in modulating immune responses and has neuroprotective properties [11], which aids in fighting against neurodegeneration induced by inflammatory reactions in neuroglial cultures [12]; it has also been shown to have antioxidant properties in neural tissue [13]. Proposed mechanism of action is mainly attributed to the reduction of NFκβ activity through inhibition of p50 subunits and preventing their binding to DNA and through disruption of binding between Keap1 and Nrf2, allowing Nrf2 to be translocated into the nucleus and to initiate the transcription of ARE-genes, which reduces inflammation and promotes neuroprotection [14, 15]. Preclinical data show that AP induces a resting phenotype in microglial cells and attenuates neurotoxicity [16, 17]; it also reduces cognitive impairment and stimulates neurogenesis in the hippocampus in adult mice [18, 19]. The beneficial effect of AP in experimental autoimmune encephalomyelitis (EAE) has been shown, in which it interferes with T cell activation and prevents EAE [20]; in a chronic EAE model, AP was shown to reduce inflammatory infiltrates and demyelination in the spinal cord [21]. Moreover, *Andrographis paniculata* standardized extract equivalent to 170 mg andrographolide daily dose showed a reduction in fatigue at 1 year in patients with relapsing-remitting MS receiving interferon beta in comparison to placebo [8].

The limited efficacy of anti-inflammatory agents in patients with PMS has been attributed to the diverse pathophysiology of the disease [4], and support the need to explore new chemical entities in specific subpopulations of PMS patients, such as older [22] or not active PMS. Since andrographolide is considered a new class of anti-inflammatory and neuroprotective agent with potential use in auto-immune diseases such as MS [14], in the present study we performed an exploratory pilot study on the efficacy of andrographolide in not active PMS patients.

## Methods

### Trial oversight

The trial was conducted by the Programa de Esclerosis Múltiple UC at the Pontificia Universidad Católica de Chile through a Corporación de Fomento (CORFO) grant (14PIE-26,946) with support from the Government of Chile and InnoBioscience SpA. The trial was designed by a protocol working group and was conducted at a single centre, and it involved a clinical trial and data coordination (ClinicalTrials.gov identifier NCT02273635). Safety monitoring and data analysis were also performed by the same group. The clinical protocol was developed and was implemented and registered in compliance with the guidelines of the Institute of Public Health of Chile (ISPCh) and the Pontificia Universidad Católica Ethics Committee, the International Council for Harmonization guidelines for Good Clinical Practice with applicable local regulations, and the ethical principles contained in the Helsinki Declaration. The study was submitted to and approved by Institute of Public Health of Chile (ISPCh) and the Pontificia Universidad Católica Ethics Committee. All the patients provided written informed consent. The active drug and matching placebo were provided by InnoBioscience SpA.

### Patients

The eligibility criteria included patients > 18 years old with a diagnosis of primary or secondary progressive MS according to McDonald 2010 criteria [23], without evidence of relapses or new T2/T1 gadolinium-enhancing lesions in the last 12 months (not active progressive MS), with or without progression, and with a baseline EDSS score < 8.0 and a Mini-Mental State Examination (MMSE) score > 24.

The exclusion criteria included patients with relapsing-remitting MS, active (clinical or radiological) primary or secondary progressive MS according to the Lublin definitions [1], the use of systemic glucocorticoid treatment within 3 months prior to screening, the use of immunomodulatory drugs or immunosuppression at least 6 months prior to screening, uncontrolled medical or psychiatric disorders, pregnancy or an inability to use effective contraception, an MMSE score < 24, and contraindications for performing an MRI.

### Trial design

Patients were recruited in a single centre at the Programa de Esclerosis Multiple UC at the Pontificia Universidad Católica de Chile in Santiago, Chile, and were assigned to receive 140 mg AP twice daily PO or an identical matched placebo tablet twice daily PO. The active and placebo coated tablets were manufactured by Laboratorios Euromed Chile S.A. according to GMP guidelines. In this study, we used a pure andrographolide compound; the dose determination was based on pre-clinical [20] and clinical trials on the use of 150–300 mg/day of AP [9, 10]. Andrographolide (99,5% purity) was provided by HPIngredients (Bradenton, FL, USA).

Forty-four subjects with not active PPMS or SPMS were enrolled, randomized and allocated in two groups (placebo and AP). Randomization was performed centrally with an Epidat 4.2 (Dirección Xeral de Saúde Pública, Xunta de Galicia, Spain) algorithm to a maximum expected sample size of *N* = 68 and two samples of equal size (*N* = 34) to generate a simple fixed-allocation randomization list (A/B). The allocation was performed by the blinded study coordinator according to the A/B list. Safety and efficacy visits were performed every 12 weeks through week 96. Extra visits due to any relevant efficacy or safety issues were allowed and were also recorded. Adherence to the trial regimen was assessed by questioning patients and counting any remaining tablets at clinical visits. Clinical disability according to the EDSS and the Multiple Sclerosis Functional Composite (MSFC) was assessed every 12 weeks by an independently trained investigator (rater) blinded to the patient group. MRI and optical coherence tomography (OCT) were performed at baseline and at month 24 of treatment. The site investigators, EDSS/MSFC raters, image analysis investigators, and patients were unaware of the trial group assignments.

MRI was performed with the same scanner for all patients. Whole brain isotropic sagittal 3D T1W MPRAGE MRI was acquired in a Philips Ingenia 1.5 T device with an 8-channel head coil. The sequence parameters were as follows: TR: 7,5 ms, TE: 3,5 ms, TI: 1000 ms, flip angle: 8°, bandwidth: 217 kHz, FOV: 240 mm, voxel size: 1 × 1 × 1 mm, number of slides: 180, SENSE: 2, NSA: 1, and scan time: 4:13 min. The two-time point percentage brain volume change was estimated with Structural Image Evaluation using Normalisation of Atrophy (SIENA) [24], which is part of FSL [25]. Analysis of the thickness of the retinal nerve-fibre layer with OCT was performed in a single reading session, and the measurements for both eyes were averaged to obtain the final result at baseline and at the end of follow-up.

### Trial endpoints

The primary endpoint was the difference in the mean percentage brain volume change, as measured by SIENA. The secondary efficacy endpoints were the percentage of patients with 12-week confirmed disability progression based on a time-to-event analysis considering an increase of 1 point when the baseline EDSS was < 5.5 and a 0.5 point increase when the baseline EDSS was > 5.5. Additional endpoints were the mean change in the retinal nerve fibre layer thickness as measured by OCT, the MSFC, the mean change in a timed 25 ft walk test (T25WT), the nine-hole peg test (9HPT), Paced Auditory Serial Addition Test (PASAT-3), the Symbol Digit Modalities Test (SDMT), the Fatigue Severity Scale (FSS), the Multiple Sclerosis Impact Scale (MSIS29), and the Beck Depression Inventory (BDI).

Safety was ensured by site investigators, who reported adverse events and serious adverse events to the local ethics committee.

### Statistical analysis

This was an exploratory pilot study designed to determine whether andrographolide is able to decrease brain atrophy after 2 years of treatment, as determined by the percentage of the change in brain volume for the treated group with respect to the non-treated group. We recruited a total of 44 participants divided in two groups (*n* = 21 for placebo and *n* = 23 for AP group).

The primary efficacy analyses were performed on data from the per-protocol analysis (including all patients who completed the two time-point MRI for brain volume analysis). The secondary efficacy analyses were performed on data from the modified intention-to treat population, which was defined as all the patients who received at least one dose of the trial regimen and had at least one efficacy assessment after the baseline [5]. Adverse events were reported based on data from all the patients who received at least one dose of the trial regimen (safety population). No imputation for missing data was performed. A general linear model was used to evaluate the differences in the mean percentage brain volume change adjusted for the baseline MSFC. The post hoc brain volume analysis included differences in the brain parenchymal fraction (BPF) change based on SIENA X normalized volumes (BPF=Normalized Grey Matter Volume + Normalized White Matter Volume / Total Intracranial Volume; BPF change = BPF (24 months) – BPF (baseline)). The three-month confirmed disability progression according to the EDSS score was analysed with the use of a two-sided log-rank test for differences between AP and the placebo, and a Cox proportional-hazards regression was used for the estimation of hazard ratios, with adjustment for baseline MSFC, sex, MS phenotype and baseline progression. To obtain the estimates of the effect of the treatment on EDSS, a linear mixed-effect model analysis was used. Incorporated data collected at scheduled visits after up to 24 months of treatment and was used to assess all data collected over time with consideration of the variance-covariance matrix of repeated measures. This method allows for the inclusion of data from patients with incomplete data at scheduled time points. The model included the comparison of EDSS scores at post baseline timepoints at each visit to that at baseline as the dependent variable. The fixed effects in the model included the independent variables of treatment and covariates such as sex and age. The between-group baseline differences were analysed with the use of Student’s t-test or a Wilcoxon rank-sum test for continuous variables and a Chi-square test or Fisher’s exact test for nominal variables. IBM SPSS Statistics 21 was used to determine the significance of results based on a two-sided *p* value < 0.05. The reporting of this study adheres to CONSORT guidelines.

## Results

### Patients

**Fig. 1:**
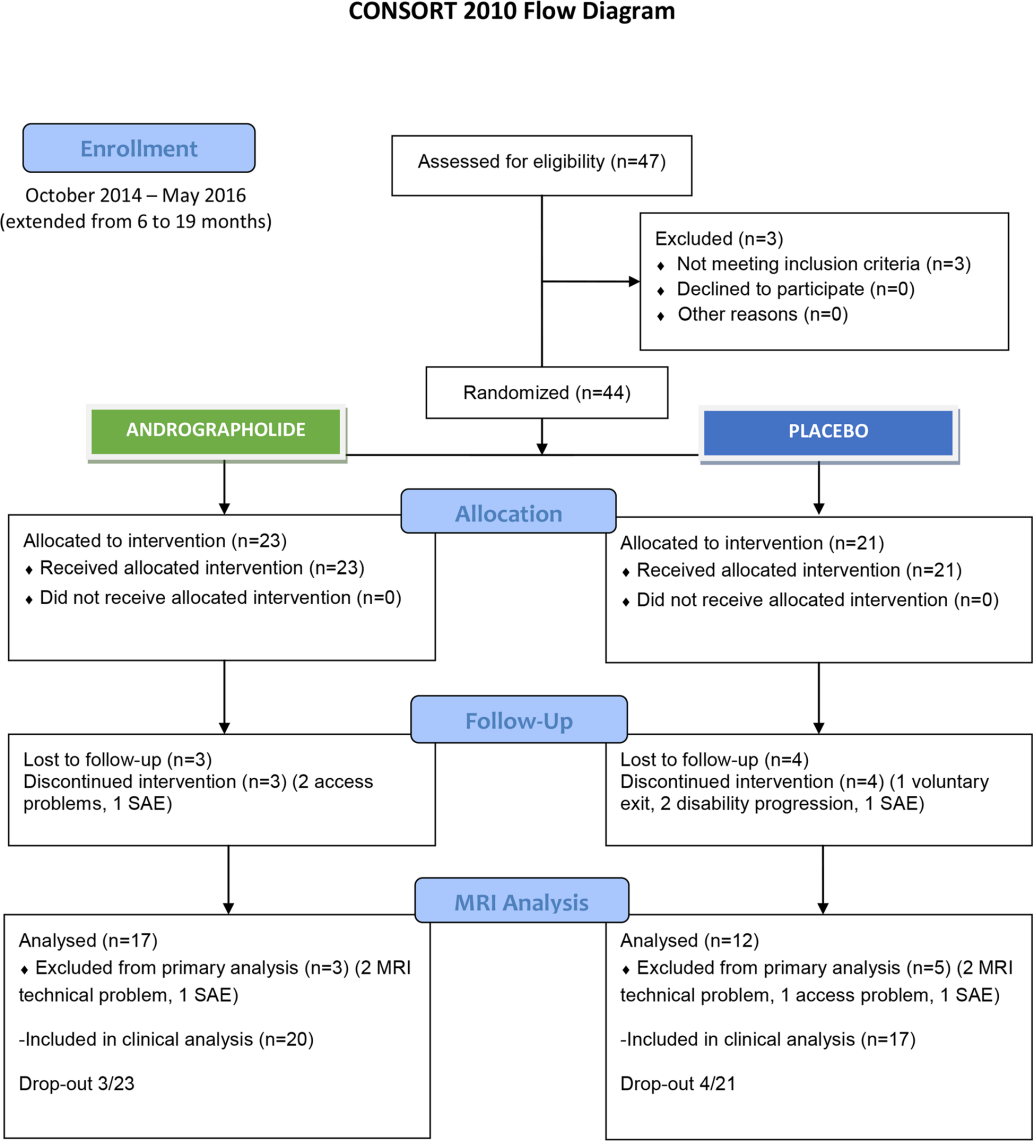
Flow chart. SAE serious adverse event, MRI magnetic resonance imaging

The expected recruiting time of 6 months was extended to 19 months from October 27th, 2014 through May 2016 due to the low recruitment rate. After 47 patients were assessed for eligibility and 3 patients were excluded, 44 patients were enrolled; 23 were assigned to the AP group, and 21 were assigned to the placebo group (Fig. 1).

The baseline demographic and clinical characteristics were similar between the two groups, except that the AP group had higher MSFC scores compared to the placebo group (median z-score − 0.14 vs − 0.01, *p* = 0.024) because 4 patients (20%) in the AP were not able to complete the T25FW at baseline compared to 1 patient (5.6%) in the placebo group. A total of 50% of the patients in the AP group and 59% in the placebo group had primary progressive multiple sclerosis (Table 1). Prior immunotherapy of included patients is available in Supplementary Material 1.

**Table 1:** Baseline demographics and clinical characteristics of included patients

Variable	Placebo ***N*** = 17	AP ***N*** = 20
**Age (years)**		
Mean ± SD	58.1 ± 7.0	59.4 ± 5.7
Median (range)	57 (50–73)	58.5 (51–70)
**Female sex, n (%)**	9 (53)	15 (75)
**Primary progressive, n (%)**	10 (59)	10 (50)
**Clinical progression in the year prior to enrolment, n (%)**	10 (59)	16 (80)
**Disease duration**		
Mean ± SD	14.2 ± 9.6	14.9 ± 11.5
Median (range)	11 (4–34)	11.5 (5–45)
**EDSS**		
Median	6	6
Range	2.5–7.0	3.5–7.0
**9-Hole Peg Test (seconds)**		
Median	23.15	20.86
Range	13.95–32.7	14.3–30.1
**Timed 25-ft walk test (seconds)**		
Median	13.47	10.48
Range	5.6–89	7.6–34.6
Not able to complete, n (%)	1 (5.6)	4 (20)
**Paced Auditory Serial Addition Test z-score**		
Mean + SD	−1.96 ± 1.48	−1.63 ± 1.57
**Multiple Sclerosis Functional Composite**		
Mean ± SD	−0.17 ± 0.59	0.20 ± 0.35
Median	−0.14	− 0.01
Range	−2.23	−0.88
**Symbol Digit Modalities Test z-score**		
Mean ± SD	−0.88 ± 0.99	0.03 ± 0.66
**Retinal nerve fibre layer thickness (μm)**		
Mean ± SD	89.97 ± 15.49	90.28 ± 12.86
**MRI activity (gadolinium-enhancing lesions), n (%)**	0 (0)	0 (0)
**Normalized brain volume (mm**^**3**^ **± SD)**	1,288,183 ± 86,721	1,311,701 ± 93,647
**Normalized white matter volume (mm**^**3**^ **± SD)**	662,732 ± 51,640	665,999 ± 48,708
**Normalized grey matter volume (mm**^**3**^ **± SD)**	625,451 ± 41,492	645,702 ± 49,755
**Brain parenchymal fraction ± SD**	0.95 ± 0.02	0.95 ± 0.02

*SD* Standard deviation. Brain Parenchymal Fraction = Normalized Brain Volume/Total Intracranial Volume. No statistically significant differences were observed between the baseline characteristics, except for the Multiple Sclerosis Functional Composite Wilcoxon rank-sum test (*p* = 0.024). Fisher’s exact test: sex, *p* = 0.16; phenotype, *p* = 0.59; baseline progression, *p* = 0.25

A total of 6 patients in the AP group and 9 patients in the placebo group were not able to undergo the 24-month MRI and were not included in the per-protocol analysis of the primary outcome. Thus, 29 patients (17 in the AP group and 12 in the placebo group) were included in the primary brain atrophy outcome analysis, and 37 patients (20 from the AP group and 17 from the placebo group) were included in the secondary efficacy analysis (3 patients from the AP group and 4 patients from the placebo group withdrew from the trial before any efficacy visit was conducted).

### Endpoint results

**Fig. 2:**
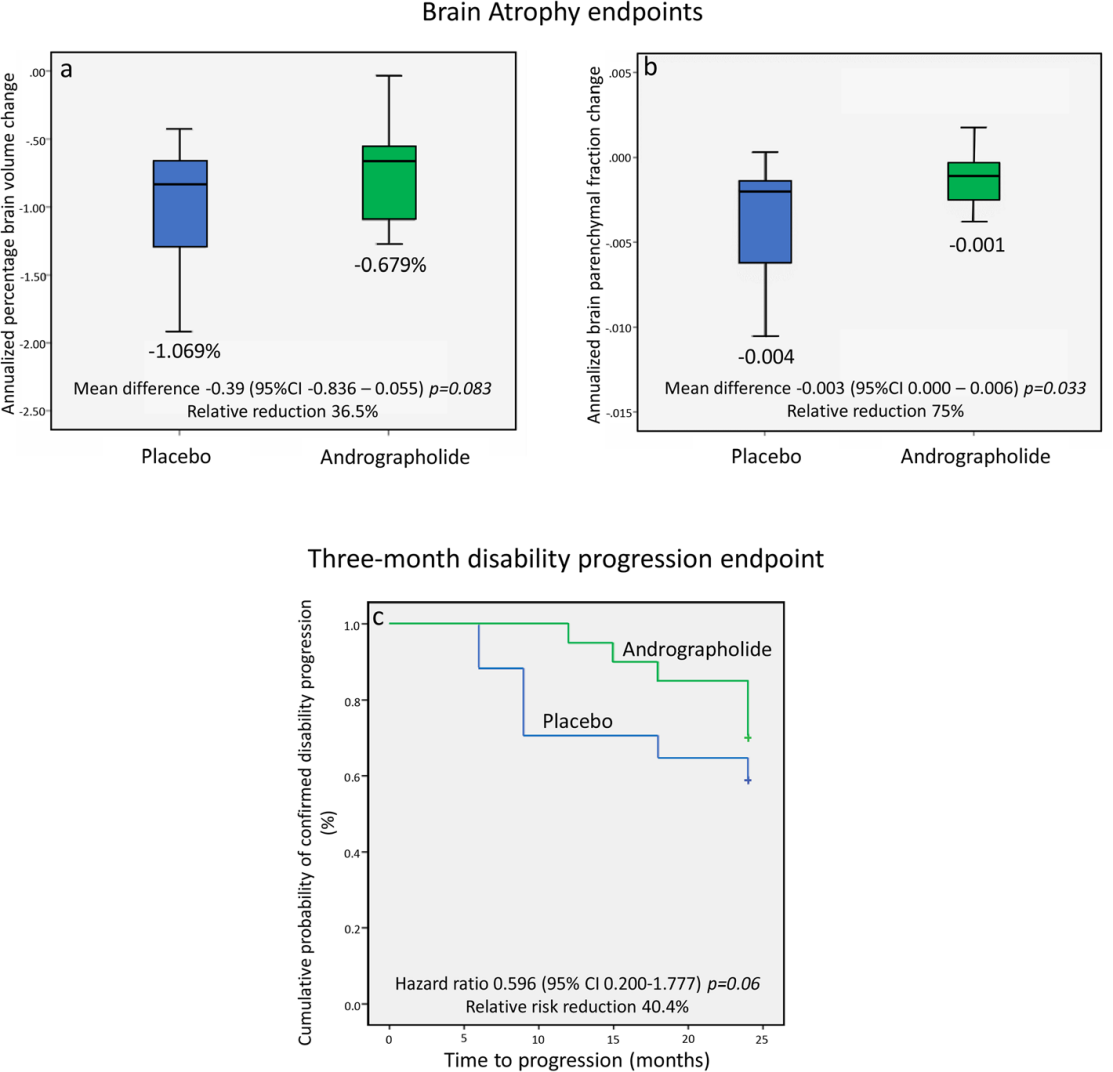
Endpoint results. *Brain Atrophy endpoints (per-protocol population).* Panel **a** shows percentage brain volume change (PBVC) as measured by SIENA comparing baseline and 24-month Magnetic Resonance Imaging (MRI). *P* value was calculated using a generalized linear model (GLM) adjusted for the baseline Multiple Sclerosis Functional Composite (MSFC). I bars indicate standard deviation. Panel **b** shows Brain Parenchymal Fraction (BPF) change comparing baseline and 24-month MRI. P value was calculated using GLM adjusted for the baseline Multiple Sclerosis Functional Composite (MSFC). I bars indicate standard deviation. *Three-month confirmed disability progression endpoint (intention-to-treat population).* Panel **c** shows cumulative probability of clinical disability progression as defined by an increase in the Expanded Disability Status Scale that was confirmed after 3 months of follow-up, in a time-to-event analysis. P value calculated with the use of the log-rank test. Cox proportional regression was used for calculation hazard ratio using baseline Multiple Sclerosis Functional Composite (MSFC), sex, phenotype and baseline progression as covariates

The estimated rate of the percentage of brain volume change was − 0.679% per year in the AP group and − 1.069% in the placebo group, which represents an absolute difference per year of − 0.39% (95% CI − 0.836 to + 0.055, *p* = 0.083) and a 35.6% relative reduction (Fig. 2a). Post hoc brain parenchymal fraction analysis showed an estimated rate of change in the AP group of − 0.001 per year compared to − 0.004 per year in the placebo group, with an absolute difference of − 0.003 (95% CI − 0.0002 to − 0.006, *p* = 0.033) and a relative reduction of 75% (Fig. 2b). Supplementary Material 2 shows subgroup analysis according to disease phenotype. By the end of the trial, 30% (6/20) of the patients in the AP group had 12-week disability progression compared to 41% (7/17) in the placebo group, with a hazard ratio of 0.596 (95% CI 0.2000–1.777) and a relative risk reduction of 40.4% (*p* = 0.06) (Fig. 2c). In the AP group, 16/20 patients had disability progression on the year prior to enrolment, compared to 6/20 by the end of the trial (*p* = 0.004). The mean EDSS change for the AP group was − 0.02 vs + 0.35 for the placebo group (*p* = 0.042). Other disability measures such as SDMT, PASAT, 9HPT, T25FW, MSFC and RNFL did not show statistical differences and are shown in Table 2.

**Table 2:** Secondary efficacy outcomes

EDSS	Placebo ***N*** = 17	AP ***N*** = 20	*p*
Baseline median EDSS	6.0 (2.5–7.0)	6.0 (3.5–7.5)	0.92
Final median EDSS	6.5 (2.0–8.0)	6.0 (3.0–7.5)	0.24
Baseline mean EDSS	5.7 ± 1.3	5.8 ± 1.1	0.93
Final mean EDSS	6.2 ± 1.3	5.8 ± 1.3	0.43
Mean EDSS change	+ 0.35 (95% CI, −0.75 - 0.05)	−0.03 (95% CI, − 0.4 - 0.4)	0.04^a^
**Other efficacy outcomes**	**Placebo*N*** **= 17**	**AP*N*** **= 20**	*p*
	mean change (95% CI)	mean change (95% CI)	
SDMT	0.49 (0.05–0.92)	0.23 (−0.13–0.59)	0.36
PASAT	0.28 (−0.57–1.09)	0.22 (− 0.44–0.89)	0.92
9HPT	0.01 (−2.18–2.16)	−1.55 (−3.65–0.54)	0.32
T25FW	1.58 (−5.39–8.56)	−1.19 (−7.15–4.77)	0.56
MSFC	0.05 (−0.19–0.29)	0.11 (− 0.62–0.29)	0.69
RNFL	−9.2 (− 12.9–5.5)	−7.3 (− 11.2–3.3)	0.47

^a^Adjusted model (baseline EDSS as a covariate); the mean difference between AP and placebo was 0.63

*SMDT*: symbol digit modalities test; *PASAT* Paced auditory serial addition test; *9HPT* Nine-hole peg test; *T25FW* Timed 25-ft walk; *MSFC* Multiple sclerosis functional composite; *RNFL* Retinal nerve fibre layer. The results from GLM were adjusted for baseline MSFC

### Safety

The percentage of patients reporting severe adverse events was 13% in the AP group and 42.8% in the placebo group, which was mainly a result of cardiovascular disease in the latter group. One death was observed in the placebo group (urinary/pulmonary sepsis), while no opportunistic infections were observed. One case of herpes simplex virus-1 encephalitis in the AP group was considered to be sporadic, and upon review, the patient’s blood sample showed no sign of immunosuppression. Adverse events that were considered to be related to AP were rash (12/23 in the AP group vs 0/21 in the placebo group) and dysgeusia (3/23 in the AP group vs 0/21 in the placebo group). One patient discontinued AP due to dysgeusia. Other adverse events were considered to be evenly distributed between the groups (Table 3). The percentage of patients who withdrew from the trial before an efficacy visit was 13% (3/20) in the AP group and 19% (4/21) in the placebo group.

**Table 3:** Adverse events recorded during treatment

Adverse Events	Placebo *N* = 21	AP *N* = 23
**Serious**		
*Infection*		
Herpes simplex virus-1 encephalitis	0	1
Urinary/pulmonary sepsis, death	1	0
Pneumonia	1	0
Influenza	1	0
Pyelonephritis	0	1
PML	0	0
*Cardiovascular*		
Acute coronary syndrome	2	1
Syncope	2	0
Pacemaker installation	2	0
*Cancer*	0	0
**Mild – Moderate**		
*Infections*		
Upper respiratory tract infection	5	8
Lower urinary tract infection	1	3
Gastrointestinal infection	2	2
*Other*		
Pruriginous rash	0	12
Dysgeusia	0	3
Falls	2	4
Mood/sleep disturbances	1	3
Gastroesophageal reflux	1	1
Lumbar pain	1	1
Joint pain	2	1
Bursitis	0	1
Cataract surgery	0	1
Gastric ulcer	1	0
Vertigo	1	0
Constipation	1	0

Comorbidities were balanced between groups (data not shown)

## Discussion

In this pilot study that included patients with not active progressive MS who suffered from a long disease duration and high levels of disability, AP showed trends in reducing brain atrophy rates and disability progression compared to placebo, which supports the potential role of andrographolide as a neuroprotective agent.

Although the primary endpoint (annualized brain percentage volume change) did not achieve statistical significance, however there was a significant relative reduction in brain atrophy in the AP group (36.5% as measured by SIENA, and 75% post hoc as measured by BPF, *p* = 0.033) compared to placebo. Recent phase 2 studies testing simvastatin [26], lipoic acid [27], ibudilast [28], and a novel multi-arm MS-SMART testing fluoxetine, amiloride or riluzole [29], have used brain atrophy as a biomarker of neurodegeneration to measure primary efficacy outcomes. In addition, our results can be compared with other progressive MS phase 2 trials, 48% of relative reduction with ibudilast [28] and 43% with simvastatin [26], also with similar rates in the placebo arms.

Three-month confirmed disability progression was observed in 30% of the AP group compared to 41% of the placebo group, which is also similar to the rates observed in larger phase 3 studies with ocrelizumab (ocrelizumab 32.9% vs placebo 39.9%) [5] and siponimod (siponimod 26% vs placebo 32%) [6]. This showed the similar histories of the placebo cohorts, even when none of the patients included in the present trial had gadolinium-enhancing lesions at baseline (27.5% in the ocrelizumab trial and 21% in the siponimod trial), and the patients were almost 10 years older and had lower baseline brain volumes than those in the phase 3 trials.

Although the differences in the other secondary efficacy endpoints were far from significant, except for the EDSS change, from a clinical point of view, it is interesting to note that in the AP group, 16/20 patients had disability progression on the year prior to enrolment, compared to 6/20 by the end of the trial (*p* = 0.004), and by the end of the trial, one patient in the AP group had recovered the ability to complete the T25FW, while in the placebo group, 4 additional patients were not able to complete the T25FW by the end of the study; this highlighted the need for better outcome measures and improved study designs for progressive MS trials.

Safety seemed comparable between the two groups, and adverse events related to the drug (dysgeusia and rash) were considered mild. Only one patient discontinued AP due to dysgeusia. Considering previous reports, mild-to moderate allergic reactions have been observed, especially with higher doses of andrographolide. In-vitro and in-vivo studies have shown that high-dose andrographolide induced histamine and LTC4 release in IgE sensitized RBL-2H3 cells, and release of tryptase, β-hexosaminidase and LTC4 tested through a non-IgE mediated pathway indicating it might induce anaphylactoid reactions [30]. Dysgeusia could be attributed to andrographolide bitterness [31]. Other adverse events described in the literature include constipation, nausea, vomiting, diarrhoea, unpleasant sensations in the chest and intensified headache, although with very low rates of presentation [10, 32].

The main limitation of the trial was the number of patients enrolled. We estimated for a decrease brain atrophy after 2 years of treatment of 1.20 units, with a standard deviation of 1.57 units [33], considering that one patient will be required for each control, to find a difference of 50% with a significance level of 0.05 and a potency of 0.80, 28 subjects in each group should be required. Longer duration of the study as well as a bigger sample size might have led to significant results. As this was mainly a University-driven exploratory study with limited resources, further alliances with larger funding entities will be required.

There were some recruitment difficulties due to restricted inclusion criteria for not active progressive MS patients, the single-centre design and the fact that the trial was conducted in a country with accessibility problems and a low prevalence of the disease, in which only relapsing-remitting MS patients had insurance coverage for diagnosis and treatment. Although some imbalances between the groups could be observed at baseline, only MSFC was statistically significant, and sensitivity analysis adjusting for other relevant variables (such as gender, or primary/secondary progression) did not show any relevant differences with the main results.

## Conclusions

Andrographolide has shown promising results in reducing brain atrophy and disability progression in not active progressive MS and has demonstrated a positive safety profile. With a proposed mechanism of action that includes anti-inflammatory and neuroprotective properties, further trials that confirm the efficacy and safety of andrographolide in larger populations or that evaluate its use in combination with other highly active disease-modifying therapies are still needed.

## Supplementary information

**Additional file 1: Supplementary Material 1**. Prior immunotherapy of included patients.

**Additional file 2: Supplementary Table 2**. Subgroup analysis for Brain Atrophy Measurements according to disease phenotype

## Data Availability

The datasets used and/or analysed during the current study are available from the corresponding author on reasonable request.

## Abbreviations

MS: Multiple sclerosis
PMS: Progressive multiple sclerosis
AP: Andrographolide
mPBVC: Mean percentage brain volume change
3-CDP: Three month confirmed disability progression
CI: Confidence interval
HR: Hazard ratio
EDSS: Expanded disability status scale
MRI: Magnetic resonance imaging
EAE: Experimental autoimmune encephalomyelitis
MMSE: Mini-mental state examination
MSFC: Multiple sclerosis functional composite
OCT: Optical coherence tomography
SIENA: Structural Image evaluation using normalisation of atrophy
T25WT: Timed 25 ft walk test
9HPT: Nine-Hole Peg Test
PASAT: Paced auditory serial addition test
SDMT: Symbol digit modalities test
FSS: Fatigue severity scale
MSIS29: Multiple sclerosis impact scale
BDI: Beck depression inventory
BPF: Brain parenchymal fraction

## References

[1] Lublin FD, Reingold SC, Cohen JA, Cutter GR, Sorensen PS, Thompson AJ (2014). Defining the clinical course of multiple sclerosis: the 2013 revisions. Neurology..

[2] Kutzelnigg A, Lassmann H (2014). Pathology of multiple sclerosis and related inflammatory demyelinating diseases. Handb Clin Neurol.

[3] Filippi M, Bar-Or A, Piehl F, Preziosa P, Solari A, Vukusic S (2018). Multiple sclerosis. Nat Rev Dis Primers.

[4] Ontaneda D, Thompson AJ, Fox RJ, Cohen JA (2017). Progressive multiple sclerosis: prospects for disease therapy, repair, and restoration of function. Lancet..

[5] Montalban X, Hauser SL, Kappos L, Arnold DL, Bar-Or A, Comi G (2017). Ocrelizumab versus placebo in primary progressive multiple sclerosis. N Engl J Med.

[6] Kappos L, Bar-Or A, Cree BAC, Fox RJ, Giovannoni G, Gold R (2018). Siponimod versus placebo in secondary progressive multiple sclerosis (EXPAND): a double-blind, randomised, phase 3 study. Lancet..

[7] Marx W, Hockey M, McGuinness AJ, Lane M, Christodoulou J, van der Mei I (2019). The effect of emerging nutraceutical interventions for clinical and biological outcomes in multiple sclerosis: a systematic review. Mult Scler Relat Disord.

[8] Bertoglio JC, Baumgartner M, Palma R, Ciampi E, Carcamo C, Caceres DD (2016). Andrographis paniculata decreases fatigue in patients with relapsing-remitting multiple sclerosis: a 12-month double-blind placebo-controlled pilot study. BMC Neurol.

[9] Hancke JL, Srivastav S, Caceres DD, Burgos RA (2019). A double-blind, randomized, placebo-controlled study to assess the efficacy of Andrographis paniculata standardized extract (ParActin(R)) on pain reduction in subjects with knee osteoarthritis. Phytother Res.

[10] Hu XY, Wu RH, Logue M, Blondel C, Lai LYW, Stuart B (2017). *Andrographis paniculata* (Chuan Xin Lian) for symptomatic relief of acute respiratory tract infections in adults and children: A systematic review and meta-analysis. PLoS One.

[11] Das S, Mishra KP, Ganju L, Singh SB (2017). Andrographolide - a promising therapeutic agent, negatively regulates glial cell derived neurodegeneration of prefrontal cortex, hippocampus and working memory impairment. J Neuroimmunol.

[12] Wang T, Liu B, Zhang W, Wilson B, Hong JS (2004). Andrographolide reduces inflammation-mediated dopaminergic neurodegeneration in mesencephalic neuron-glia cultures by inhibiting microglial activation. J Pharmacol Exp Ther.

[13] Chern CM, Liou KT, Wang YH, Liao JF, Yen JC, Shen YC (2011). Andrographolide inhibits PI3K/AKT-dependent NOX2 and iNOS expression protecting mice against hypoxia/ischemia-induced oxidative brain injury. Planta Med.

[14] Tan WSD, Liao W, Zhou S, Wong WSF (2017). Is there a future for andrographolide to be an anti-inflammatory drug? Deciphering its major mechanisms of action. Biochem Pharmacol.

[15] Tavakkoli A, Iranshahi M, Hasheminezhad SH, Hayes AW, Karimi G (2019). The neuroprotective activities of natural products through the Nrf2 upregulation. Phytother Res.

[16] Chan SJ, Wong WS, Wong PT, Bian JS (2010). Neuroprotective effects of andrographolide in a rat model of permanent cerebral ischaemia. Br J Pharmacol.

[17] Yang R, Liu S, Zhou J, Bu S, Zhang J (2017). Andrographolide attenuates microglia-mediated Abeta neurotoxicity partially through inhibiting NF-kappaB and JNK MAPK signaling pathway. Immunopharmacol Immunotoxicol.

[18] Serrano FG, Tapia-Rojas C, Carvajal FJ, Hancke J, Cerpa W, Inestrosa NC (2014). Andrographolide reduces cognitive impairment in young and mature AbetaPPswe/PS-1 mice. Mol Neurodegener.

[19] Varela-Nallar L, Arredondo SB, Tapia-Rojas C, Hancke J, Inestrosa NC (2015). Andrographolide stimulates neurogenesis in the adult Hippocampus. Neural Plast.

[20] Iruretagoyena MI, Tobar JA, Gonzalez PA, Sepulveda SE, Figueroa CA, Burgos RA (2005). Andrographolide interferes with T cell activation and reduces experimental autoimmune encephalomyelitis in the mouse. J Pharmacol Exp Ther.

[21] Tichauer JME, Acuña E, Burgos R, Hancke J, Carcamo C, Naves R. Andrographolide suppresses chronic and relapsing-remitting experimental autoimmune encephalomyelitis. In: Front Immunol Conference Abstract: 15th International Congress of Immunology (ICI) doi: 103389/conffimmu20130200301. 2013. https://www.frontiersin.org/10.3389/conf.fimmu.2013.02.00301/event_abstract.

[22] Vaughn CB, Jakimovski D, Kavak KS, Ramanathan M, Benedict RHB, Zivadinov R (2019). Epidemiology and treatment of multiple sclerosis in elderly populations. Nat Rev Neurol.

[23] Polman CH, Reingold SC, Banwell B, Clanet M, Cohen JA, Filippi M (2011). Diagnostic criteria for multiple sclerosis: 2010 revisions to the McDonald criteria. Ann Neurol.

[24] Smith SM, Zhang Y, Jenkinson M, Chen J, Matthews PM, Federico A (2002). Accurate, robust, and automated longitudinal and cross-sectional brain change analysis. Neuroimage..

[25] Smith SM, Jenkinson M, Woolrich MW, Beckmann CF, Behrens TE, Johansen-Berg H (2004). Advances in functional and structural MR image analysis and implementation as FSL. Neuroimage..

[26] Chataway J, Schuerer N, Alsanousi A, Chan D, MacManus D, Hunter K (2014). Effect of high-dose simvastatin on brain atrophy and disability in secondary progressive multiple sclerosis (MS-STAT): a randomised, placebo-controlled, phase 2 trial. Lancet..

[27] Spain R, Powers K, Murchison C, Heriza E, Winges K, Yadav V (2017). Lipoic acid in secondary progressive MS: A randomized controlled pilot trial. Neurol Neuroimmunol Neuroinflamm.

[28] Fox RJ, Coffey CS, Conwit R, Cudkowicz ME, Gleason T, Goodman A (2018). Phase 2 trial of Ibudilast in progressive multiple sclerosis. N Engl J Med.

[29] Chataway J, De Angelis F, Connick P, Parker RA, Plantone D, Doshi A (2020). Efficacy of three neuroprotective drugs in secondary progressive multiple sclerosis (MS-SMART): a phase 2b, multiarm, double-blind, randomised placebo-controlled trial. Lancet Neurol.

[30] Richard EJ, Murugan S, Bethapudi B, Illuri R, Mundkinajeddu D, Chinampudur VC (2017). Is Andrographis paniculata extract and andrographolide anaphylactic?. Toxicol Rep.

[31] Zhang X, Wu H, Yu X, Luo H, Lu Y, Yang H (2018). Determination of bitterness of Andrographis Herba based on electronic tongue technology and discovery of the key compounds of bitter substances. Molecules..

[32] Li F, Li H, Luo S, Ran Y, Xie X, Wang Y (2018). Evaluation of the effect of andrographolide and methotrexate combined therapy in complete Freund's adjuvant induced arthritis with reduced hepatotoxicity. Biomed Pharmacother.

[33] Altmann DR, Jasperse B, Barkhof F, Beckmann K, Filippi M, Kappos LD (2009). Sample sizes for brain atrophy outcomes in trials for secondary progressive multiple sclerosis. Neurology..

