# Supplementary material for: Efficacy of andrographolide in not active progressive multiple sclerosis: a prospective exploratory double-blind, parallel-group, randomized, placebo-controlled trial
Source: BMC Neurol. 2020 May 7;20:173. doi: 10.1186/s12883-020-01745-w (PMC7203851; doi:10.1186/s12883-020-01745-w)
Supplement: Supplementary file 1 — Additional file 1: Supplementary Material 1. Prior immunotherapy of included patients. [file 12883_2020_1745_MOESM1_ESM.docx]

**Supplementary Material 1. Prior immunotherapy of included patients**.

| **Immunotherapy suspended at least 6 months prior to screening** | **Placebo N=21** | **AP N=23** |
| --- | --- | --- |
| Never received  Interferon-beta 1a IM  Interferon-beta 1a SC  Interferon-beta 1b SC  Glatiramer Acetate  Teriflunomide  Azathioprine  Mitoxantrone  Cyclophosphamide  Intravenous Immunoglobulin  Rituximab | 12  5  1  1  3  0  1  3  1  1  3 | 12  6  0  1  4  2  2  1  0  1  0 |

| **Placebo Group** | | **AP Group** | |
| --- | --- | --- | --- |
| 1 | - | 1 | - |
| 2 | - | 2 | - |
| 3 | IFNb 1a IM | 3 | IFNb 1a IM |
| 4 | - | 4 | - |
| 5 | - | 5 | - |
| 6 | - | 6 | - |
| 7 | - | 7 | - |
| 8 | - | 8 | TER |
| 9 | IFNb 1a IM, MTX, RTX | 9 | AZA |
| 10 | - | 10 | IFNb 1b SC |
| 11 | IVIG, IFNb 1a IM, GA, MTX, CYC, RTX | 11 | IFNb 1a IM, GA, MTX |
| 12 | MTX, IFNb 1a IM | 12 | IFNb 1a IM, GA |
| 13 | IFNb 1a SC | 13 | IFNb 1a IM, GA |
| 14 | GA | 14 | - |
| 15 | IFNb 1b SC | 15 | AZA |
| 16 | GA, RTX, AZA | 16 | IFNb 1a IM, GA |
| 17 | IFNb 1a IM | 17 | TER |
| 18 | - | 18 | - |
| 19 | - | 19 | - |
| 20 | - | 20 | - |
| 21 | - | 21 | - |
|  |  | 22 | IFNb 1a IM, IVIG |
|  |  | 23 | - |
| IFNb 1a IM: interferon beta 1a intramuscular; IFNb 1a SC: interferon beta 1a subcutaneous; IFNb 1b SC: interferon beta 1b subcutaneous; GA: glatiramer acetate; AZA: azathioprine; MTX: mitoxantrone; RTX: rituximab; CYC: cyclophosphamide; IVIG: intravenous immunoglobulin; TER: teriflunomide | | | |
