# Supplementary material for: Efficacy of andrographolide in not active progressive multiple sclerosis: a prospective exploratory double-blind, parallel-group, randomized, placebo-controlled trial
Source: BMC Neurol. 2020 May 7;20:173. doi: 10.1186/s12883-020-01745-w (PMC7203851; doi:10.1186/s12883-020-01745-w)
Supplement: Supplementary file 2 — Additional file 2: Supplementary Table 2. Subgroup analysis for Brain Atrophy Measurements according to disease phenotype [file 12883_2020_1745_MOESM2_ESM.docx]

**Supplementary Table 2. Subgroup analysis for Brain Atrophy Measurements according to disease phenotype**

| **Brain Atrophy Measurements** | **Placebo (n=12)** | **AP**  **(n=17)** | **Mean change (95%CI)** | **p** |
| --- | --- | --- | --- | --- |
| *Annualized Percentage Volume Change* |  |  |  |  |
| Primary Progressive MS | -1.067 (n=7) | -0.667 (n=8) | -0.4 (-1.16 to +0.36) | 0.26 |
| Secondary Progressive MS | -1.084 (n=5) | -0.681 (n=9) | -0.4 (-1.18 to +0.37) | 0.27 |
| *Brain Parenchymal Fraction Change* |  |  |  |  |
| Primary Progressive MS | -0.003 (n=7) | -0.002 (n=8) | -0.001 (-0.005 to +0.004) | 0.74 |
| Secondary Progressive MS | -0.005 (n=5) | -0.0001 (n=9) | -0.005 (-0.009 to -0.0003) | 0.037 |
| AP andrographolide; GLM Adjusted by baseline Multiple Sclerosis Functional Composite (MSFC) | | | | |
